# Supplementary material for: Adolescents’ Exposure to Online Risks: Gender Disparities and Vulnerabilities Related to Online Behaviors
Source: Int J Environ Res Public Health. 2021 May 27;18(11):5786. doi: 10.3390/ijerph18115786 (PMC8199225; doi:10.3390/ijerph18115786)
Supplement: Supplementary file 1 [file ijerph-18-05786-s001.zip › ijerph-1215643-supplementary.pdf]

International Journal of
Environmental Research and Public Health

Supplementary Material A: Survey Instrument

HARVARD
T.H. CHAN
SCHOOL OF PUBLIC HEALTH
Emergency Preparedness Research,
Evaluation, and Practice Program

If you intend to use this questionnaire for your project, please cite the publication and inform the authors by sending an e-mail to preparedness@hsph.harvard.edu.

Q1 Select your grade

- o 8
- o 9

Q2 What have most of your grades been up to now at this school?

- o A
- o A-, B+
- o B
- o B-, C+
- o C or Lower

Q3 What is your age?

- o 12
- o 13
- o 14
- o 15
- o 16

Q4 What gender do you identify with?

- o Male
- o Female
- o Rather Not Say
- o Other. Please specify: ____________

Q5 What race/ethnicity do you consider yourself? Please select as many as you see fit:

- ☐ American Indian or Alaska Native
- ☐ Arab
- ☐ African American

- ◻ Native Hawaiian or other Pacific Islander
- ◻ Non-Hispanic White
- ◻ Non-Hispanic Black
- ◻ Asian
- ◻ East Asian
- ◻ Central Asian
- ◻ Western Asian
- ◻ Southeast Asian
- ◻ South Asian
- ◻ Haitian
- ◻ Hispanic
- ◻ Somali
- ◻ Don't know
- ◻ Rather not say
- ◻ Other. Please specify: ____________

Q6 Which of the following social media tools do you use?

Copyright: © 2021 by the authors. Licensee MDPI, Basel, Switzerland. This article is an open access article distributed under the terms and conditions of the Creative Commons Attribution (CC BY) license (http://creativecommons.org/licenses/by/4.0/).

Social Media Tool	How often do you use the social media tool?
Twitter	o All the time<br>o Several times a day<br>o 1–2 times per day<br>o Several times per week<br>o Several times per month<br>o Never
Facebook	o All the time<br>o Several times a day<br>o 1–2 times per day<br>o Several times per week<br>o Several times per month<br>o Never
Google+	o All the time<br>o Several times a day<br>o 1–2 times per day<br>o Several times per week<br>o Several times per month

	o Never
LinkedIn	o All the time<br>o Several times a day<br>o 1–2 times per day<br>o Several times per week<br>o Several times per month<br>o Never
YouTube	o All the time<br>o Several times a day<br>o 1–2 times per day<br>o Several times per week<br>o Several times per month<br>o Never
Salesforce Chatter	o All the time<br>o Several times a day<br>o 1–2 times per day<br>o Several times per week<br>o Several times per month<br>o Never
Skype	o All the time<br>o Several times a day<br>o 1–2 times per day<br>o Several times per week<br>o Several times per month<br>o Never
Tango	o All the time<br>o Several times a day<br>o 1–2 times per day<br>o Several times per week<br>o Several times per month<br>o Never
MySpace	o All the time<br>o Several times a day<br>o 1–2 times per day<br>o Several times per week<br>o Several times per month<br>o Never
Digg	o All the time<br>o Several times a day<br>o 1–2 times per day<br>o Several times per week<br>o Several times per month

	o Never
Flickr	o All the time<br>o Several times a day<br>o 1–2 times per day<br>o Several times per week<br>o Several times per month<br>o Never
Reddit	o All the time<br>o Several times a day<br>o 1–2 times per day<br>o Several times per week<br>o Several times per month<br>o Never
Instagram	o All the time<br>o Several times a day<br>o 1–2 times per day<br>o Several times per week<br>o Several times per month<br>o Never
Pinterest	o All the time<br>o Several times a day<br>o 1–2 times per day<br>o Several times per week<br>o Several times per month<br>o Never
Snapchat	o All the time<br>o Several times a day<br>o 1–2 times per day<br>o Several times per week<br>o Several times per month<br>o Never
I use other social media tool(s)<br>Please enter the name below:<br>____________________________	o All the time<br>o Several times a day<br>o 1–2 times per day<br>o Several times per week<br>o Several times per month<br>o Never

Q7 Do you use private messaging apps? Check all that apply

- ☐ WhatsApp
- ☐ Kik
- ☐ Telegram

- ◻ I do not use private messaging apps
- ◻ Other. Please specify: _____________

Q8 Do you use private messaging apps more than texting?

- ○ Yes
- ○ No
- ○ Not sure

Q9 Think of your social media account that has the most connections. Below, please choose the answer that best shows how many connections on this account you know and have met in person.

- ○ Some of them
- ○ Most of them
- ○ All of them
- ○ I am not sure how many
- ○ I don't have a social media account

Q10 Do you ever chat/ communicate with friends or individuals on social media whom you don't know (have never met in person)?

- ○ Never
- ○ I did it a few times
- ○ Sometimes
- ○ Often
- ○ I don't have a social media account

Q11 To the best of your knowledge, do your friends chat/ communicate with individuals on social media whom they don't know (have never met in person)?

- ○ Never
- ○ Sometimes
- ○ Often
- ○ I don't know

Q12 When you post on social media, how often do you mention or tag the town you live in, your school's name, your location, or other personal information?

- ○ Never
- ○ Sometimes
- ○ Often
- ○ I don't have a social media account

Q13 Do you play with multiplayer gaming systems? (XBox, Playstation, PC gaming, etc.)

- ○ Never
- ○ Sometimes
- ○ Often

Q14 Do you ever chat/ communicate with players while gaming that you don't know?

- o Never
- o Sometimes
- o Often
- o I do not play video games

Q16 Has anyone ever talked to you about online safety?

- o Never
- o Sometimes
- o Often

Q17 Have you ever experienced any of the following situations online? Select all that apply.

- ☐ Bullying or harassment by friends or acquaintances
- ☐ Getting involved in unwanted conversations in a chat room, social networking site or on e-mail
- ☐ Coming across sexual images or content
- ☐ Coming across images of violence
- ☐ Someone trying to sell me drugs or alcohol
- ☐ Someone using my photos in an inappropriate way
- ☐ A stranger trying to meet with me
- ☐ Coming across hate groups trying to convince me of their views
- ☐ None of these has happened to me
- ☐ Other. Please describe: _____________

Q18 Do you feel you have a trusted adult to ask for help with the situations in the previous questions?

- o Yes
- o No
- o Not sure
- o It depends on the situation. Please explain: _____________

Q19 Has anyone ever sent you messages or pictures that made you feel uncomfortable?

- o Yes
- o No

Q20 Have your parents set up parental controls on your computer or other devices?

- o Yes
- o No
- o Not sure

Supplementary Material B: Simple Logistic Regression Models

Covariate	OS 1 Bullying	OS 2 Unwanted conversations	OS 3 Sexual images	OS 4 Violent images	OS 5 Drugs & alcohol	OS 6 Photos used inappropriately	OS 7 Stranger	OS 8 Hate Groups	At least one unsafe event
**Continuous and Dichotomous Predictors—OR (95% CI)**									
Age (15 and older vs. 14)	1 (0.7–1.4)	1.2 (0.8–1.7)	1.1 (0.8–1.5)	1.4 (1–2) *	1.3 (0.8–2)	0.8 (0.4–1.5)	1.2 (0.8–1.8)	1.2 (0.8–1.9)	1.3 (0.9–1.7)
Gender (Female vs. Male)	1.8 (1.3–2.4) **	2 (1.4–2.9) **	2.1 (1.5–2.9) **	2 (1.5–2.8) **	1.5 (1–2.3)	2.1 (1.1–4.4) *	2.5 (1.6–3.7) **	1.6 (1–2.5) *	2.1 (1.5–2.8) **
Race (White vs. Non-White)	1.2 (0.8–1.6)	1 (0.7–1.4)	1.4 (1–1.9)	1.1 (0.8–1.6)	1.1 (0.7–1.7)	0.8 (0.4–1.6)	0.7 (0.5–1)	0.9 (0.6–1.4)	1.1 (0.8–1.4)
Grades (B and lower vs. B+ and higher)	1 (0.7–1.4)	1 (0.7–1.4)	1.3 (0.9–1.8)	1.5 (1–2.1) *	0.9 (0.5–1.4)	0.9 (0.5–1.9)	0.7 (0.5–1.2)	1.4 (0.9–2.2)	1.2 (0.8–1.6)
Kik Use (Yes vs. No)	2.4 (1.2–4.7)*	1.6 (0.8–3.3)	2.8 (1.5–5.5) **	1.3 (0.7–2.6)	2.5 (1.2–5.3) *	4.4 (1.8–10.7) **	2.5 (1.2–5.1) *	3.2 (1.6–6.7) **	2.5 (1.1–5.5) *
WhatsApp Use (Yes vs. No)	1.3 (0.8–2.2)	1.1 (0.6–1.9)	1.3 (0.8–2.2)	1.3 (0.8–2.2)	1.4 (0.7–2.7)	1.7 (0.7–4.2)	1.9 (1.1–3.4) *	1.6 (0.8–3)	1.7 (1–3) *
Telegram Use (Yes vs. No)	0.7 (0.1–3.3)	2.4 (0.6–8.9)	0.6 (0.1–3.1)	1.1 (0.3–4.2)	0.8 (0.1–6.3)	4.9 (1–24.3)	1.4 (0.3–6.8)	3.2 (0.8–12.8)	2.3 (0.5–11.3)
Chatting with strangers on social media (Any Frequency of Communication vs. Never/I don't have a social media account)	2.2 (1.5–3.1) **	2.5 (1.7–3.7) **	1.9 (1.4–2.7) **	2 (1.5–2.9) **	2.5 (1.5–4.1) **	6.3 (2.2–17.8) **	2.2 (1.4–3.4) **	2.3 (1.4–3.8) **	2.9 (2.1–3.9) **
Sharing Personal Information on Social Media (Any Frequency of Sharing vs. Never/I don't have a social media account)	1.2 (0.8–1.6)	1.6 (1.1–2.3) *	1.3 (0.9–1.8)	1.3 (0.9–1.8)	1.4 (0.9–2.1)	2.7 (1.4–5) **	1.7 (1.2–2.6) *	1.2 (0.8–1.9)	1.8 (1.3–2.5) **
Playing video games (Any Frequency of Use vs. Never)	0.8 (0.5–1.1)	0.9 (0.6–1.4)	0.7 (0.5–1)	0.8 (0.6–1.1)	1 (0.6–1.7)	0.6 (0.3–1.2)	0.6 (0.4–0.9) *	1 (0.6–1.7)	0.7 (0.5–1)
Chatting with strangers while playing video games (Any Frequency of Communication vs. Never/I don't play video games)	1 (0.7–1.3)	1 (0.7–1.4)	0.8 (0.6–1.1)	0.9 (0.6–1.2)	1.2 (0.8–1.9)	0.9 (0.5–1.6)	0.5 (0.3–0.7) **	1 (0.6–1.5)	0.8 (0.6–1.1)
Having someone talk to you about online safety (continuous)	1.25 (0.94–1.64)	1.17 (0.87–1.56)	1.16 (0.89–1.53)	1.39 (1.06–1.83) *	1.24 (0.85–1.8)	0.87 (0.52–1.46)	1.41 (1–2.01)	1.41 (0.96–2.06)	1.34 (1.05–1.73) *
Social Media Use (continuous)	1.27 (1.14–1.41) **	1.28 (1.15–1.43) **	1.29 (1.16–1.43) **	1.31 (1.18–1.46) **	1.33 (1.16–1.53) **	1.33 (1.09–1.63) **	1.19 (1.05–1.35) *	1.22 (1.06–1.39) **	1.33 (1.21–1.48) **
**Categorical Predictors with 3+ Categories—Chi-Squared (df); *p*-value**									
Use Private Messaging Apps More than Texting (categorical)	2.54 (2); $p = 0.281$	2.34 (2); $p = 0.31$	1.13 (2); $p = 0.569$	3.45 (2); $p = 0.178$	4.62 (2); $p = 0.099$	3.19 (2); $p = 0.203$	4.17 (2); $p = 0.125$	3.17 (2); $p = 0.205$	2.51 (2); $p = 0.285$
Amount of followers known in person (categorical)	7.48 (4); $p = 0.113$	12.63 (4); $p = 0.013$	16.88 (4); $p < 0.01$	15.11 (4); $p < 0.01$	12.13 (4); $p = 0.016$	7.65 (4); $p = 0.106$	8.78 (4); $p = 0.067$	9.96 (4); $p = 0.041$	18.65 (4); $p < 0.01$
Frequency that friends chat	5.16 (3); $p = 0.161$	15.6 (3); $p < 0.01$	18.35 (3); $p < 0.01$	21.39 (3); $p < 0.01$	14.01 (3); $p < 0.01$	1.22 (3); $p = 0.748$	12.11 (3); $p =$	15.14 (3); $p < 0.01$	28.56 (3); $p<0.01$

with strangers on social media (categorical)							0.007		
Having a trusted adult to ask for help with unsafe online situations (categorical)	7.58 (3); $p = 0.056$	7.07 (3); $p = 0.07$	6.75 (3); $p = 0.08$	5.58 (3); $p = 0.134$	5.54 (3); $p = 0.136$	8.05 (3); $p = 0.045$	2.79 (3); $p = 0.425$	1.46 (3); $p = 0.691$	6.33 (3); $p = 0.097$
Presence of parental controls on your computer (categorical)	1.33 (2); $p = 0.513$	0.82 (2); $p = 0.663$	1.92 (2); $p = 0.384$	1.53 (2); $p = 0.465$	1.65 (2); $p = 0.438$	2.64 (2); $p = 0.267$	2.11 (2); $p = 0.348$	2.6 (2); $p = 0.273$	1.39 (2); $p = 0.499$

* $p < 0.05$; ** $p < 0.01$; OS 1, bullying or harassment by friends or acquaintances; OS 2, getting involved in unwanted conversations in a chat room, social networking site or on email; OS 3, coming across sexual images or content; OS 4, coming across images of violence; OS 5, someone trying to sell me drugs or alcohol; OS 6, someone using my photos in an inappropriate way; OS 7, a stranger trying to meet with me; OS 8, coming across hate groups trying to convince me of their views.
